# Analysis of clozapine prescribing in the over-65s: 5-year retrospective study

**DOI:** 10.1192/bjb.2024.55

**Published:** 2025-10

**Authors:** James Barclay, Rahul Tomar, Tim Gale

**Affiliations:** Hertfordshire Partnership NHS Foundation Trust, Hatfield, UK

**Keywords:** Antipsychotics, polypharmacy, psychotic disorders/schizophrenia, drug or substance interactions and side-effects, life expectancy

## Abstract

**Aims and method:**

Patients prescribed clozapine are increasingly living into old age. However, there is a lack of studies to guide prescribing in this age group. We sought to identify all clozapine patients in Hertfordshire Partnership NHS Foundation Trust over a 5-year period and review side-effect burden and co-prescribing in all patients aged over 65 years.

**Results:**

We identified 69 patients. The majority (61%) were stable in terms of mental state; 94% of cases had experienced a side-effect within the past year, with constipation occurring most commonly (65% of cases).

**Clinical implications:**

Our findings reveal a significant side-effect burden, particularly in relation to constipation. Clozapine-induced gastrointestinal hypomotility (CIGH) can be fatal; however, increasing age has not been a recognised risk factor for constipation in clozapine patients to date. This raises questions about increasing risk to physical health as patients age and adds to concerns about the lack of monitoring for CIGH.

Clozapine is the only effective evidence-based treatment for treatment-resistant schizophrenia,^1^ and this is reflected in the clinical guidelines.^2^ However, prescribing is limited in all age groups by the wide range of adverse effects. Some of these are acute medical concerns, for example, myocarditis, seizures and agranulocytosis (with attendant blood-monitoring requirements), whereas others such as metabolic syndrome, hypersalivation and sedation are more troubling on a day-to-day basis. Constipation can be both.^3^

Of the population in the UK, 18.6% is aged 65 years or older,^4^ and many patients with a schizophrenia diagnosis are living into old age. Elderly patients are particularly susceptible to the adverse effects of antipsychotic drugs, including clozapine, because of age-related physiological changes that can alter the pharmacodynamics and pharmacokinetics of drugs. Concurrent comorbidities and drug interactions are also more likely. Prescribing considerations relevant to elderly patients include reviewing the risk of falls related to co-prescription of anti-hypertensives, opioids and benzodiazepines. Anticholinergic burden is also commonly assessed, owing to its recognised contribution to cognitive impairment and potentially fatal constipation. However, despite these concerns, there is limited research to guide recommendations for the use of clozapine in this age group.^5^

## Aims

We identified all patients over 65 years old in Hertfordshire Partnership NHS Foundation Trust (HPFT) who had been prescribed clozapine over a 5-year period. The objectives were (a) to assess side-effect burden; (b) to review concurrent medications, focusing on psychotropics and medications that may contribute to side-effects; and (c) to ascertain the relationship between side-effects and clozapine dose.

## Method

We undertook a retrospective analysis of electronic patient records for all patients who had been prescribed clozapine within HPFT over the preceding 5 years (2018–2022). Data were collected from the Denzapine Monitoring Service (DMS) and HPFT's Information Team. Registration with DMS is mandatory; therefore, it is very unlikely that any patients were missed. Those over the age of 65 years were identified. Case note entries, clinic letters, discharge summaries and any side-effect scales used were reviewed in detail. Where patients had died or discontinued use of clozapine during the 5-year period, we reviewed these data for the preceding year.

Ethics approval was not required as this was an audit using retrospective, routinely collected clinical data. The manuscript is an honest, accurate, and transparent account of the study being reported and no important aspects of the study have been omitted.

## Results

### Demographic details

We identified a total of 508 patients, 13% (*n* = 69) of whom were over the age of 65 years. The mean patient age was 70 years; 54% were male and 46% female. From most to least common, the diagnoses were schizophrenia (74%; *n* = 51), schizoaffective disorder (22%; *n* = 15), psychosis in Parkinson's disease (3%; *n* = 2) and depression with psychotic symptoms (1.4%; *n* = 1).

### Treatment commencement and dosage

The majority (80%) of prescriptions were started before 65 years of age. The mean combined total daily dose was 301 mg. This dose was higher (332 mg total mean daily dose) if prescribing was started before the age of 65 and lower if prescribed after the age of 65 (150 mg total mean daily dose).

### Mental health stability and side-effect burden

Sixty-one per cent (*n* = 42) of patients had been stable in their mental health (relapse/crisis intervention) for at least 5 years, as evidenced by not requiring crisis intervention or in-patient treatment; 29% (*n* = 20) had relapsed within 5 years, and a further 10% (*n* = 7) were unwell at the point of data collection.

Ninety-four per cent (*n* = 65) of patients had experienced side-effects within the past year, or in the year before discontinuation/death. As noted in Table 1, constipation was the most commonly recorded side-effect, occurring in 65% (*n* = 45) of cases.

**Table 1:** Side-effects noted

Side-effects within past year (most to least common)	Number of patients	%
Constipation	45	65
Hypersalivation	25	36
Falls	16	23
Postural hypotension	6	9
Sedation	11	16
Dizziness	4	6
Seizure	3	4
Extrapyramidal side-effects	2	3
Neutropenia	1	1
Nil noted	4	6

### Co-prescribing

Twenty per cent (*n* = 14) of patients were prescribed an additional antipsychotic (Table 2). Of these, 50% (*n* = 7) were prescribed amisulpride, 14% (*n* = 2) aripiprazole, 7% (*n* = 1) haloperidol, 7% (*n* = 1) quetiapine, 7% (*n* = 1) risperidone, 7% (*n* = 1) sulpride and 7% (*n* = 1) a zuclopenthixol decanoate depot.

**Table 2:** Co-prescribing noted

Co-prescriptions (most to least common)	Number of patients	%
Antidepressants	23	33
Additional antipsychotic	14	20
Mood stabiliser (excluding lithium)	14	20
Antihypertensives	13	19
Hyoscine	11	16
Benzodiazepine/z-drug	7	10
Lithium	5	7
Pregabalin	2	3
Acetylcholinesterase inhibitor	1	1
Opioid	1	1
Nil	8	12

An average score of 4.5 was noted using the Anticholinergic Effect on Cognition scoring system, where a score of 3+ is associated with cognitive impairment and mortality.^6^

### Constipation and clozapine dosage

As constipation was the most common side-effect, we attempted to ascertain whether any association existed between clozapine dose and constipation. The patients with constipation noted were prescribed a mean combined daily dose of 323 mg, whereas the group without constipation noted were prescribed a mean combined daily dose of 260 mg. This difference was not statistically significant (*t*[67] = 1.67, *P* = 0.099). However, the *P*-value was small enough that we could consider the possibility of a type II error due to sample size.

#### Discontinued group

Of the 69 patients in total, seven patients’ clozapine was discontinued during the 5-year period. Their mean age was 74 years. Two of these were receiving end-of-life care, and clozapine discontinuation was a planned component of their management. In the remaining five cases, discontinuation was due to side-effects. Clozapine was discontinued owing to constipation or pseudo-obstruction in two cases, and because of seizures, sedation and myocarditis in one case each.

#### Deceased group

Nine patients died while taking clozapine, with a mean age of 71 years. In none of the cases was clozapine considered to be the cause of death, and all deaths were due to natural causes.

## Discussion

A survey of old age psychiatrists showed that consultant psychiatrists felt that clozapine had a useful place in treatment of elderly patients, but they were concerned about the lack of published data on its safety.^7^ Recognised concerns include clozapine's strong anticholinergic properties, which may contribute to cognitive impairment and potentially fatal constipation; the risk of hypersalivation, which may predispose to aspiration pneumonia;^5^ and the elevated risk of cerebrovascular events when prescribing antipsychotics in this age group.^8^

More generally, older people have additional risk factors for developing constipation.^3^ They are more likely to have reduced mobility, they may have reduced ability to express pain, they are likely to be co-prescribed analgesics or other drugs that can cause constipation, and they are more sensitive to anticholinergic effects of drugs compared with their younger counterparts.^9^

Our study reveals a group of clozapine patients over the age of 65 who have maintained a relatively stable mental state over a 5-year period. However, we have also demonstrated a significant side-effect burden. The main concern emerging from the data relates to the high rate of constipation reported (65%). Related to this, the data also highlight concerns about co-prescribing of medications with anticholinergic properties. The constipation rate is substantially higher than estimates for individuals of 65 years of age or older in the community, where the prevalence is estimated to be 26% for women and 16% for men.^10^ It is also higher than an estimated prevalence of 31% for clozapine-associated constipation across age groups, derived from a meta-analysis of 32 studies.^11^ In clinical practice, patients frequently fail to report side-effects when asked openly about them, whereas systematic inquiry gives a more accurate picture.^12^ Therefore, documentation in clinical records is likely to be an underestimation of the true side-effect burden.

There is little in the existing literature to suggest that increasing age is a risk factor for constipation in patients prescribed clozapine. A large prospective database study of 26 720 patients with schizophrenia identified an association between increasing age and increased risk of ileus, where the average age of ileus patients was 56 years and that of controls was 47 years.^13^ This study was not specific to clozapine, however. Our study therefore presents a novel finding, suggesting that constipation may be an underrecognised and underreported complaint in this age group.

Patients with schizophrenia diagnoses tend to have pre-existing risk factors for constipation, such as reduced dietary fibre, weight gain, a sedentary lifestyle and dehydration.^11^ The mechanism by which clozapine causes constipation is not fully understood; however, clozapine's strong anticholinergic properties lead to gastrointestinal hypomotility, inhibiting normal gut peristalsis. This may lead to low stool frequency, lack of urge to defecate, abdominal distension, bloating and abdominal discomfort.^14^ This clozapine-induced gastrointestinal hypomotility (CIGH) can contribute to a multifactorial process leading to ileus, pseudo-obstruction and gastrointestinal ischaemia, and it can prove fatal.

Concerns are emerging that insufficient attention has been paid to CIGH. This may be in part because of its non-specific presentation: constipation is the most commonly reported problem, but it can also present as dysphagia, bowel obstruction, ‘acute abdomen’ or faecal aspiration. There is no simple, accurate diagnostic test, in contrast to agranulocytosis, which is a discrete condition easily diagnosed with a blood test.^15^ Recent epidemiological studies suggest that mortality due to CIGH is at least three times greater than that of agranulocytosis.^13,16^ Moreover, coroners alerted the health secretary in 2018 to two deaths related to clozapine side-effects, amid concerns that healthcare staff may not be sufficiently aware of the drug's serious side-effects.^17^ In 2017, a coroner highlighted lack of awareness of gastrointestinal effects such as pseudo-obstruction, alongside the rapid onset of these effects when they do occur. Furthermore, a less serious degree of chronic constipation not amounting to serious hypomotility has been consistently shown to diminish quality of life.^18^

Opinions differ about the relationships among clozapine dose, plasma levels, and rates of CIGH or ileus. A subgroup analysis in a 22-year binational pharmacovigilance study in Australia and New Zealand suggested that age, female gender, dose and receiving other constipating medications have positive but non-significant associations with fatal outcomes due to CIGH.^19^ Meanwhile, a review of the literature lists higher doses as risk factors for CIGH.^20^ The same review showed that in cases where deaths have occurred as a result of CIGH, higher than average daily doses were prescribed (mean 532 mg per day). Our study is therefore consistent in suggesting an association between increasing dose and constipation incidence, although this association does not reach statistical significance.

An area of upcoming focus should be developing strategies for preventing and managing CIGH and the broader side-effect burden in elderly clozapine users. One practical approach that we suggest deserves consideration is an annual clozapine plasma level from the age of 65. There is evidence that mean plasma concentrations may increase after the age of 65 despite lower clozapine dosage.^21^ Furthermore, adverse effect monitoring may not occur as regularly over time, and there may not be timely reviews of clozapine dose requirement.^15^ People also tend to smoke less as they get older,^22^ which could have a marked effect on dose requirement. An annual review of plasma levels would highlight those at risk of adverse effects and may in turn prompt a dosage review.

The number of chronic conditions and co-prescribed medications tends to increase with age, and structured medication reviews with a focus on potential deprescribing are therefore indicated. There is a particular risk that anticholinergic medications such as hyoscine, used to treat hypersalivation, may exacerbate CIGH.^15^ The co-prescription of medications known to increase the risk of constipation, such as opioids, should be minimised.^11^ There are existing resources for use in older adults, such as the STOPPFrail tool,^23^ which specifically highlights classes of medicines for review in frail patients with limited life expectancy, for whom medicines with long-term preventive effects are likely to be less beneficial.

More broadly, there are several principles for reducing the risk of CIGH based on current guidance. Education for clozapine users and family/carers is recommended, with particular emphasis on seeking medical attention for the ‘red flags’ of moderate-to-severe abdominal pain lasting more than an hour, distended abdomen, vomiting, diarrhoea with blood in it or feeling unwell. Regular monitoring is indicated, and use of the Glasgow Antipsychotics Side-effects Scale is advised because it specifically asks about constipation.^24^ Finally, a recent review highlights the role of routine use of prophylactic laxatives, alongside an emphasis on modifiable risk factors, namely actively increasing fibre intake, increasing fluid intake and increasing physical activity where possible.^25^

In conclusion, our study raises concerns about the incrementally increasing risk to physical health in an ageing group of clozapine patients. It also raises questions as how to best address risk–benefit discussions in the absence of large-scale studies or guidelines for this age group. There are well-established systems of blood monitoring for agranulocytosis; however, no such monitoring procedures currently exist for constipation. This is despite evidence suggesting that mortality due to CIGH is at least three times greater than that caused by agranulocytosis. This study raises particular concerns regarding the high rate of constipation in elderly clozapine users, and we suggest that particular attention should be paid to this. In light of these concerns, prescribers are advised to consider an annual clozapine level and related dosage review.

## Data Availability

The data that support the findings of this study are available from the corresponding author, J.B., upon reasonable request.

## About the authors

**James Barclay** is an ST6 dual general-older adult trainee at Hertfordshire Partnership NHS Foundation Trust, Hatfield, UK. **Rahul Tomar** is a consultant old age psychiatrist at Hertfordshire Partnership NHS Foundation Trust, Hatfield, UK. **Tim Gale** is Research and Development lead, Hertfordshire Partnership NHS Foundation Trust, Hatfield, UK.
